# *In vivo* Two-Photon Imaging Reveals Acute Cerebral Vascular Spasm and Microthrombosis After Mild Traumatic Brain Injury in Mice

**DOI:** 10.3389/fnins.2020.00210

**Published:** 2020-03-10

**Authors:** Xinjia Han, Zhi Chai, Xingjie Ping, Li-Juan Song, Cungen Ma, Yiwen Ruan, Xiaoming Jin

**Affiliations:** ^1^Spinal Cord and Brain Injury Research Group, Stark Neurosciences Research Institute, Indiana University School of Medicine, Indianapolis, IN, United States; ^2^Department of Anatomy and Cell Biology, Indiana University School of Medicine, Indianapolis, IN, United States; ^3^Department of Obstetrics and Gynecology, Guangzhou Women and Children’s Medical Center, Guangzhou Medical University, Guangzhou, China; ^4^GHM Institute of CNS Regeneration (GHMICR), Jinan University, Guangzhou, China; ^5^Neurobiology Research Center, Shanxi Key Laboratory of Innovative Drugs for Serious Illness, College of Basic Medicine, Shaanxi University of Chinese Medicine, Jinzhong, China; ^6^Co-innovation Center of Neuroregeneration, Nantong University, Nantong, China

**Keywords:** cerebral cortex, vasculature, blood circulation, mild traumatic brain injury, two-photon imaging

## Abstract

Mild traumatic brain injury (mTBI), or concussion, is reported to interfere with cerebral blood flow and microcirculation in patients, but our current understanding is quite limited and the results are often controversial. Here we used longitudinal *in vivo* two-photon imaging to investigate dynamic changes in cerebral vessels and velocities of red blood cells (RBC) following mTBI. Closed-head mTBI induced using a controlled cortical impact device resulted in a significant reduction of dwell time in a Rotarod test but no significant change in water maze test. Cerebral blood vessels were repeatedly imaged through a thinned skull window at baseline, 0.5, 1, 6 h, and 1 day following mTBI. In both arterioles and capillaries, their diameters and RBC velocities were significantly decreased at 0.5, 1, and 6 h after injury, and recovered in 1 day post-mTBI. In contrast, decreases in the diameter and RBC velocity of venules occurred only in 0.5–1 h after mTBI. We also observed formation and clearance of transient microthrombi in capillaries within 1 h post-mTBI. We concluded that *in vivo* two-photon imaging is useful for studying earlier alteration of vascular dynamics after mTBI and that mTBI induced reduction of cerebral blood flow, vasospasm, and formation of microthrombi in the acute stage following injury. These changes may contribute to early brain functional deficits of mTBI.

## Introduction

Traumatic brain injury (TBI) is increasingly being recognized as a significant problem of public health. Mild traumatic brain injury (mTBI), or concussion, which accounts for 70–90% of the TBI patient population, often occurs in falls, motor vehicle accidents and sports-related injury (Cassidy et al., 2004; McCrory et al., 2005; Chiu et al., 2007). Although mTBI is known to cause chronic post-concussive syndrome with symptoms such as memory and thinking impairments, headache, anxiety, and sleep disorders that significantly affect the quality of life and long-term outcomes, the injury rarely causes specific neurological deficits or brain structural damages that are detectable in clinical imaging (Levin et al., 1987; Bramlett and Dietrich, 2004; McCrory et al., 2009). This lack of evidence of brain structural and functional damage represents a major challenge in understanding the mechanism of this disease and finding effective therapeutics (Margulies, 2000; Boake et al., 2005).

The cerebral vasculature consists of a complicated network of arteries, veins and capillaries, and plays an essential role in sustaining energy supply and metabolism of the cortex by delivering glucose and oxygen (Sunwoo et al., 2011; Shih et al., 2012a). Abnormal cerebral circulation is known to occur not only in patients with moderate to severe TBI but also in patients of mTBI during the acute and chronic stages (Bonne et al., 2003; Werner and Engelhard, 2007; Len and Neary, 2011). A strong correlation exists between microvascular dysfunction and TBI outcome, supporting that damage to the microvasculature may be a key contributor to secondary complications, including ischemia and increased intracranial pressure (Golding et al., 1999). Studies in animal models of mTBI found that pathophysiological alterations of cerebral vasculature include increase or decrease in cerebral blood flow, disruption of blood brain barrier, endothelial damage, and loss or altered vascular reactivity to various vasodilators (DeWitt et al., 1986; Wei et al., 2009; Zweckberger et al., 2010; Buckley et al., 2015). However, little information is available about acute dynamic changes in brain vessels and circulation after mTBI, particularly at the microscopic level. Given the critical importance of cerebral perfusion and oxygenation to normal brain function and its role in subsequent secondary injury and recovery, understanding cerebral microcirculation at the acute stage will be fundamental for understanding the mechanism of mTBI and its short- and long-term outcomes.

The recent two-photon imaging technique provides a powerful tool for studying longitudinal changes in cerebral vasculature and blood flow after brain ischemia and brain injuries (Schaffer et al., 2006; Drew et al., 2010a). To better mimic clinical situation of concussion in which most patients have no skull fracture or brain penetration and no positive findings in their neuroimaging examination (Alexander, 1995; McCrory et al., 2009), we used a closed-head mTBI preparation for *in vivo* two-photon longitudinal imaging of the cerebral vasculature and for revealing potential pathological changes in cerebral blood flow. Our results showed that mTBI resulted in decreases in the diameters of cerebral blood vessels as well as the velocities of red blood cells (RBCs), which is attributable to reduced cerebral blood flow and microthrombosis in capillaries.

## Materials and Methods

### Animals

Male C57BL/6J mice or the same background Thy1-YFP transgenic mice were used in this study. For imaging, mice at the ages between 8 and 10 weeks old were divided into a sham group (11 mice) and an mTBI group (15 mice). The animals were kept on a 12 h light/dark cycle with sufficient food and water. The experiment was performed according to a protocol approved by the Institutional Animal Care and Use Committee (IACUC) of the Indiana University School of Medicine.

### Thinned-Skull Window Preparation

**FIGURE 1:**
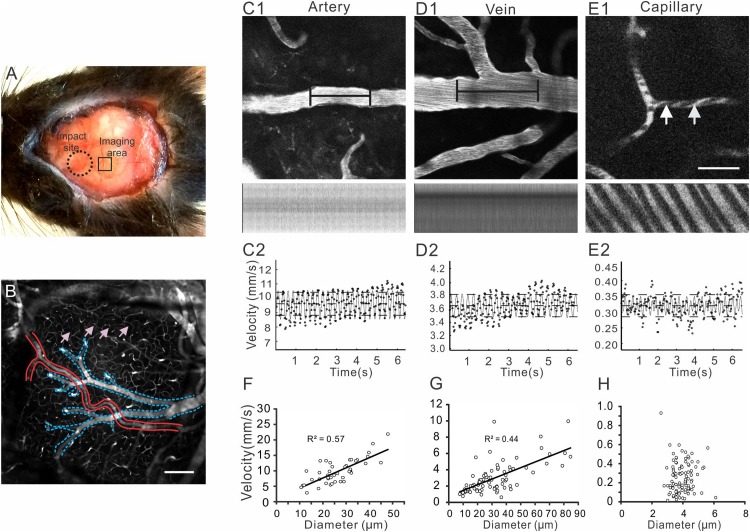
*In vivo* two-photon microscopy for imaging cerebral vasculature and measuring blood flow through a thinned-skull window. **(A)** The skull above left parietal cortex of a mouse was thinned and strengthened by gluing a piece of square coverglass (∼ 1.5 × 1.5 mm) for *in vivo* imaging (small square). Closed-head mTBI was induced by using a controlled cortical impact (CCI) device with a 3 mm diameter tip to strike on an area that was about 1 mm rostral to the anterior edge of the imaging window (large dotted circle). **(B)** A representative image of Z series projection of cerebral vessels reveals arteries (delineated in red), veins (delineated in blue), and networks of capillaries (pink arrows). **(C–E)** A segment of arteriole **(C1)**, venule **(D1)**, or capillary **(E1)** was oriented in a horizontal direction (top row) and imaged in line scan mode at 1 ms/line, which generated dark stripes (second row). The measurements were obtained from a single vessel. Velocity of red blood cells (RBC) in each vessel was calculated based on the line scan using a Matlab script **(C2–E2)**; the resulting velocities at different moments (small dots in **C2–E2**) were fitted to a second-order Fourier series (oscillating solid lines); the dashed horizontal lines represented time-averaged velocities. **(F–H)** Analyses of the relationships between RBC velocities and vessel diameters revealed positive correlations in arterioles and venules **(F,G)**, but not in capillaries **(H)**. Scale bars in B and E1 for C1-E1: 50 μm.

Reinforced thinned-skull imaging windows were prepared based on a technique described previously (Drew et al., 2010b; Shih et al., 2012b). The mice were anesthetized with an intraperitoneal (i.p.) injection of ketamine/xylazine (87.7/12.3 mg/kg), and the scalp skin was removed to expose the skull. A 2 × 2 mm skull thinning area was prepared on the left parietal cortex, with the rostral edge being 2 mm posterior to the bregma and medial edge being 2–3 mm lateral from the middle line (Figure 1A). At the beginning of the surgery, a microdrill was used to thin a 1–2 mm diameter circular skull region to about a half of the thickness, then a 10^#^ surgical blade was used to slowly and carefully thin the skull until surface blood vessels on the cerebral cortex were clearly visible under a light microscope. During this process, 0.9% physiological saline was added to the skull surface from time to time to reduce heat. After the thinned skull became dry, a small drop of thin cyanoacrylate glue (Ted Pella, Inc., Cat# 1003) was applied and a small piece of coverglass (1–1.5 × 1–1.5 mm size) was placed onto the thinned skull. The remaining area of the skull was covered with a layer of cyanoacrylate glue. The mice were allowed to recover for least 2 days before starting imaging sessions.

### Preparation of Closed-Head mTBI

Following initial imaging of cerebral vasculature to record baseline conditions, a mouse model of closed-head mTBI was created on the left hemisphere by using a controlled cortical impact device, modified from a previously described technique (Creed et al., 2011). After the mice were anesthetized with ketamine/xylazine (87.7/12.3 mg/kg, i.p.) injection, their heads were stabilized on a stereotaxic apparatus by using a pair of ear rods with the tips enclosed in a piece of sponge. To avoid rigidly holding the head, the incisor hook of the stereotaxic device was not used. The skull orientation was adjusted to make sure it was in a horizontal position and perpendicular to the impactor tip. A 3 mm diameter impactor tip was used to strike the head at 5.0 m/s to a depth of 1.0 mm with a dwell time of 10 ms (Creed et al., 2011). The posterior edge of the impactor tip was targeted to an area ∼1 mm rostral to the anterior edge of the imaging window (Figure 1A). The mice of the sham injury group were subjected to the same procedures without receiving an impact. All animals were allowed to recover on a heating pad and were returned to their home cages after recovering from anesthesia. Of 13 and 20 mice of the sham and mTBI groups, 2 and 5 mice were used for *in vivo* imaging, respectively due to poor quality of cranial windows.

### Behavioral Testing

To determine the effect of mTBI on cognitive and motor functions in this model, another set of mice were randomly assigned to a sham group (*n* = 12) and a mild TBI group (*n* = 10).

#### Rotarod Test

Motor coordination was assessed with a Rotarod apparatus (IITC Life Science Inc., CA, United States). Mice were placed on a spinning cylindrical rod accelerating from 0 to 30 rpm and the time to fall off was recorded. The maximum latency if a mouse did not fall was 120 s. Before injury, each mouse received three training trials per day with inter-trial intervals of 20–30 min, and the latency to fall on the third day was recorded as baseline (day 0). Then the mice were split randomly into the sham and the mild TBI groups, and were tested on days 2, 4, 6, 8, and 10 after sham or mild TBI surgery. The mean latencies to fall off the Rotarod at each time point were used for result analysis.

#### Morris Water Maze

The water maze was performed in a circular tank (110 cm in diameter) filled with 22°C water, which was made opaque by adding non-toxic white paint. A hidden platform (10 cm in diameter) was placed in the southwestern quadrant 1 cm below the water surface. External cues for spatial reference were attached on the inner walls of the tank. Mice were released into the tank from a random starting point and trained to find the hidden platform for four trials per day. The maximum time for each trial was 60 s. Mice that didn’t find the platform within 60 s were manually guided to it and allowed to sit on the platform for 30 s. Escape latency, and swimming distance and velocity were recorded by automated tracking software TopScan (CleverSys). Animals were trained on days 1, 3, 5, 7, and 9 after sham or mild TBI surgery, and the average latency to reach the platform on each training day was computed by averaging the four trials.

### *In vivo* Two-Photon Imaging and Measurements of Red Blood Cell (RBC) Velocity and Blood Vessel Diameters

The mice were anesthetized with ketamine/xylazine (87.7/12.3 mg/kg, i.p.) injection, and received an i.p. injection of 0.1 ml Rhodamine B sothiocyanate-dextran solution (70 KDa, 3 mg/ml in distilled water) at least 15 min before imaging to label blood plasma. The mice were placed on a miniature stereotaxic apparatus and the head position was adjusted so that the cortical surface was horizontal to the microscope objective. The mice were placed on a heating pad to maintain their body temperature, and the arterial blood pressure and heart rate were monitored via the tails using a non-invasive Volume Pressure Recording (VPR) system (CODA Surgical Monitor, Kent Scientific Corporation, CT). To obtain consistent measurements of blood pressure, the animals were placed in position for 5 min prior to obtaining pressure measurements and the tail cuff was positioned to the base of the tail. At each time, at least three measurements were made for each mouse for calculating mean values of diastolic and systolic pressures.

Imaging was made with a two-photon microscope (Ultima Multiphoton Imaging System, Bruker, United States) (Xiong et al., 2017). Excitation was provided by a Maitai diode laser source pumping a Tsunami Ti: sapphire laser (Spectra-Physics, Mountain View, CA, United States) tuned to 870 nm wavelength. Band-pass-filtered fluorescence (560–660 nm) was collected by photo-multiplier tubes of the Prairie View Ultima system. Images (512 × 512 pixels, 0.15 μm/pixel in the *x*- and *y*-axes) or line scans were acquired using Prairie View software (Figures 1C–E). The mice were imaged at baseline, and at 0.5, 1, 6 h, and 1 day after mTBI. A Z-series of blood vessel images within 100–200 μm of the pial surface were first captured under a 10X objective. Then an Olympus SUPER 20X water immersion objective was used to image individual blood vessels and take line scan images along the central axis of a vessel at 3X optical zoom. The orientation of a segment of blood vessel (∼35 μm) was adjusted so that it paralleled the direction of the line scan. Typically, each session of line scan consisted of 5000 lines with a spatial resolution of 0.7 μm per pixel and at a speed of about 1 ms per line. The line scan parameters were automatically recorded in the Prairie View software.

For longitudinal imaging, the same imaging fields and segments of line-scanned vessels at different time points after initial baseline imaging were identified based on the location and patterns of vessels. The depth and orientation of the vessels were carefully adjusted so that the same vessels were imaged with the same imaging parameters including pixel size and scanning speed. Each experimental session took 20–30 min. From baseline to 0.5 and 1 h imaging, anesthesia and labeling of blood plasma were carried out only once. For 6 h and 1 day imaging sessions, anesthesia and labeling of blood plasma were done separately. The animals were allowed to recover after each imaging on a heating pad and then returned to their home cages after being awake. Images of microthrombosis were captured in two mice after mTBI. Because the microthrombosis was transient and sporadic, the RBC velocities and diameters of the involved capillaries were not measured.

**FIGURE 2:**
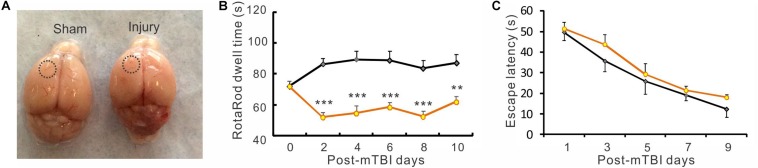
Closed-head mTBI resulted in impaired motor function. **(A)** Morphology of freshly dissected mouse brains from sham injured (left) and mTBI (right) mice. There was no obvious difference between them in gross brain structure. The dotted circles indicate approximate regions of sham or actual cortical impact. **(B)** In repeated RotaRod tests, the dwell times of the mTBI group were highly significantly less than those of the sham group between days 1 and 10 post-mTBI (F_(1, 100)_ = 106.47, **p < 0.01, ***p < 0.001, two-way ANOVA followed by Bonferroni post-test), suggesting an impaired motor function in these mice. **(C)** The escape latency in water maze test had no significant change after mTBI. (all *p* > 0.05. *n* = 12 in sham and *n* = 10 in mTBI).

Imaging analyses were made with Metlab and NIH ImageJ software. Maximal intensity projections of image stacks were made for identifying and confirming imaged vasculature in regions of interest. RBC velocities were calculated from line-scan images using a Metlab script written according to previous work of line-scanning particle image velocimetry developed by Kim et al. (2012). Briefly, this technique determines RBC displacements between pairs of line-scans using spatial cross-correlation analysis. The shift from the origin to the peak center of the cross-correlation was a measure of the distance traveled by RBCs between image frames. After the peak was fitted with a Gaussian distribution to improve calculation accuracy, the shift in pixels was converted to microns and velocity was calculated. The resulting velocities at different moments were fitted to a second-order Fourier series (Figures 2C2–E2), time-averaged velocities were obtained (Figures 2C2–E2). The diameters of line-scanned blood vessels were determined in ImageJ by manually measuring the maximal widths at the middle of the scanned vessel segment that was filled with fluorescence dye. For each vessel at each time point, three measurements were made and averaged. For measuring vessel diameters at different time points, efforts were taken to make diameter measurements at the same spots as close as possible.

### Statistics

The data were averaged among animals in each group and all data are presented as mean ± standard error of the mean (SE). Because of the large variability of the imaged vessels in size and its corresponding blood flow, vessel diameters and RBI velocities are expressed as percent change from baseline. For comparisons of diameters and velocities at different time points between the sham and mTBI groups, data were analyzed with a two-way repeated measures ANOVA using Origin Pro 9.1 software, followed by pairwise comparisons using Bonferroni test. A *p* value less than 0.05 (*p* < 0.05) was considered statistically significant. Graphs of the data were obtained using GraphPad Prism 5 software.

## Results

### Imaging Cerebral Blood Vessels Through a Thinned-Skull Window *in vivo*

In this *in vivo* imaging study, we used a thinned skull window preparation, because preparing these windows were less invasive (without exposing the brain) and they usually sustained the physical impacts generated from a CCI device during mTBI model preparation. Table 1 summarized numbers of measured vessels and numbers of animals in each experimental group. Occasionally, mice with damaged or deteriorating windows were excluded from further experiment and data analysis. As shown in Figure 1A, the posterior edge of an impact site was about 1 mm from the anterior edge of an imaging window, which would allow us to observe changes in cerebral vessels after the relatively diffuse brain injury (Drew et al., 2010b; Shih et al., 2012b).

**TABLE 1:** The number of imaged vessels and animals for each experimental group.

Group	Artery		Vein		Capillary	
	mice	vessels	mice	vessels	mice	vessels
Diameter and velocity relationship	19	44	21	79	16	104
Sham group	11	29	11	54	11	49
mTBI group	15	19	15	41	15	48

Cerebral vessels including arteries, capillaries, and veins were clearly visible within ∼400 μm below the pial surface (Figure 1B). The fluorescence dye (Rhodamine B) revealed the serum in bright red and the RBCs as dark dots due to their impermeability to the dye. RBC velocities of different vessels, which typically represent the velocities of blood flow, were measured using line scan and calculated from the dark strips (Figures 1C–E; Drew et al., 2010a; Shih et al., 2012a). The mean velocity at baseline was calculated by combining data from the shame group and mTBI groups. Arterials were differentiated from veins based on their smaller sizes, smoother vessel walls, fewer branches, and faster RBC velocities. RBC velocities of most arteries were between 5 and 20 mm/s, the velocities of veins ranged between 1 and 10 mm/s, and the velocities of capillaries were less than 1 mm/s with a mean value of 0.25 ± 0.02 mm/s.

We analyzed the relationships between vessel diameters and RBC velocities in arteries, veins, and capillaries. As expected, there were positive correlations between RBC velocities and diameters of arteries and veins, with the correlation coefficients being 0.57 and 0.44 for arteries and veins, respectively (Figures 1F,G). In contrast, the diameters of capillaries had no correlation with RBC velocities (Figure 1H).

### mTBI Induced Motor Deficit but Did Not Impair Learning and Memory Function

Closed-head mTBI induced by the modified CCI technique produced little gross brain structure damage. We observed freshly dissected mouse brains and found that closed-head mTBI induced by the modified CCI technique produced little gross brain structure damage (Figure 2A). However, behavioral tests revealed that the mTBI model produced motor behavioral deficits, as indicated by a significant reduction of dwell time in a Rotarod test in the mTBI group than the sham group during a 10-day period after injury (Figure 2B. *p* < 0.01 or *p* < 0.001, two-way ANOVA followed by Bonferroni post-test). In contrast, in the water maze test there was no significant difference in the latency of finding the hidden platform between the two groups (Figure 2C, *p* > 0.05), indicating that this type of mTBI preparation didn’t significantly impair spatial learning and memory in the mice.

### No Significant Changes in Arterial Blood Pressure After mTBI

**FIGURE 3:**
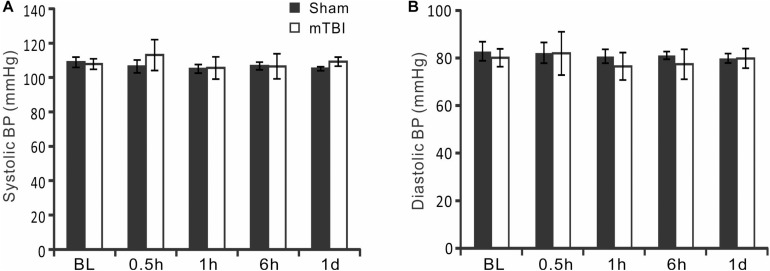
Measurement of systematic blood pressures. **(A,B)** Blood pressure (BP) was monitored by measuring tail artery BP throughout the duration of the imaging experiment. There were no significant changes in systolic BP and diastolic BP at baseline and different times after mTBI (*p* > 0.05, repeated measure ANOVA).

To monitor whether mTBI affected systemic blood pressure, systolic blood pressure (SBP) and diastolic blood pressure (DBP) of mouse tail were measured in the sham and injury groups using a tail cuff. Repeated measurements were carried out in each animal at each imaging time point (Figures 3A,B). In the sham group, the SBP and DBP were between 105.1 ± 2.5 and 108.9 ± 3.0 mmHg and between 79.9 ± 2.0 and 82.8 ± 4.0 mmHg, respectively. In the mTBI group, the SBP and DBP were between 107.9 ± 3.0 and 113.0 ± 9.0 mmHg and between 76.5 ± 5.8 and 81.9 ± 9.1 mmHg, respectively. There were no significant differences between the two groups at different time points (all *p* > 0.05).

### Significant Decreases in Diameters and RBC Velocities of Arterioles After mTBI

**FIGURE 4:**
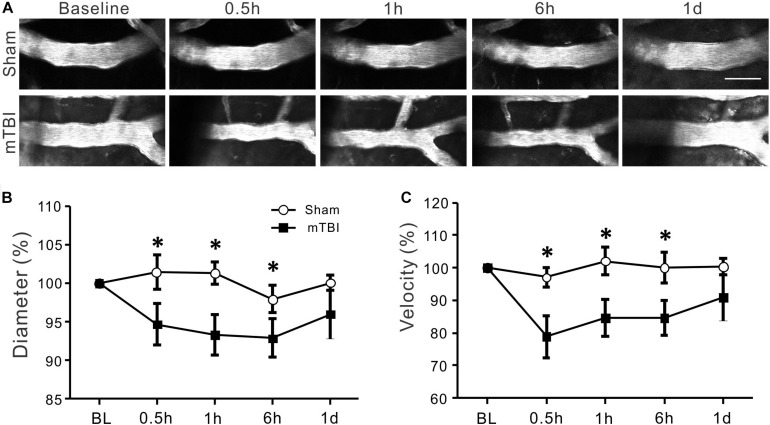
Reduced arterial diameter and RBC velocity after mTBI. **(A)** Changes in cerebral arterial morphology at different time points after mTBI in sham and injury groups. In comparison to the sham group, arteries of the mTBI group showed obvious constriction between 0.5 and 6 h post-mTBI. **(B)** Arterial diameters became smaller between 0.5 and 6 h post-injury (*p* < 0.05 at all time points when compared with the same time points of the sham groups, repeated measure ANOVA), and partially recovered to baseline level at one day post-mTBI (*p* > 0.05). **(C)** Consistent with the reduced diameter, arterial RBC velocity also significantly decreased between 0.5 and 6 h and recover to thebaseline level at 1 day following mTBI (*p < 0.05).

Morphological changes of arterioles at different time points in sham and mTBI groups are shown in Figure 4A. In the sham group, arterioles maintained stable morphology overtimes. However, spasm of arterioles was markedly visible at 0.5, 1, and 6 h following closed-head mTBI. Quantitative analysis showed that normalized arterial diameters were 101% ± 2, 101% ± 1, 97% ± 2, and 100% ± 1 at 0.5, 1, 6 h, and 1 day in the sham group (Figure 4B, *p* > 0.05). However, the diameter of arterioles decreased significantly at 0.5, 1, and 6 h post-injury (94% ± 3, 93% ± 3, and 93% ± 2, respectively, all *p* < 0.05 when compared with the sham group) and partially recovered at 1 day after mTBI (96% ± 3, *p* > 0.05 when compared with the sham group). The RBC velocities of the sham group were 97% ± 3, 102% ± 4, 100% ± 5, 100% ± 3 at 0.5, 1, 6 h, and 1 day, respectively. It was also significantly reduced at 30 min, 1, and 6 h after injury (Figure 4C 79% ± 6, 84% ± 5, and 84% ± 5, respectively, all *p* < 0.05 when compared with the sham group) and recovered partially at 1 day following mTBI (91% ± 4, *p* > 0.05 when compared with the sham group). The data suggest that both the diameter and blood flow of arterials decreased significantly during 0.5–6 h after mTBI and partially recovered in 1 day after mTBI.

### Acute Reduction but Earlier Recovery in Diameter and RBC Velocity of Venules After mTBI

**FIGURE 5:**
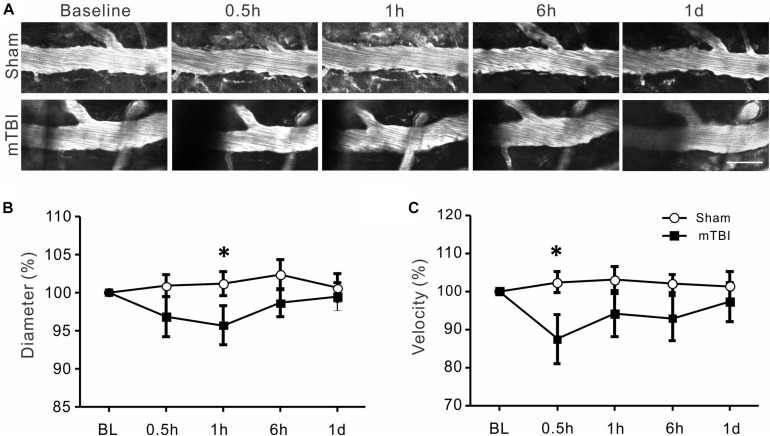
Changes in venous diameter and RBC velocity after mTBI. **(A)** Representative images of cerebral veins at different time points after mTBI of the sham and injury groups. There seems a small decrease in vein diameter within 0.5–1 h post-mTBI, which recovered to baseline level in one day. **(B)** There was a significant decrease in venous diameter at 1 h after mTBI (*p* < 0.05), but insignificant changes at other time points (*p* > 0.05). **(C)** The changes in venous RBC velocity were similar with the changes in vein diameter: a significant decrease in venous RBC velocity occurred at 0.5 h (*p* < 0.05) post-mTBI, which recovered in 1 h to 1 day post-mTBI. *p < 0.05.

The morphology of small veins remained relatively stable at different time points in both sham group and injury group (Figure 5A). Quantitative analysis revealed that both diameter and velocity of venules underwent significant reduction in diameter at 1 h (95% ± 3, *p* < 0.05) and in velocity at 30 min (87% ± 6, *p* < 0.05) after mTBI when compared with the sham group at the corresponding time points (Figures 5B,C). However, these two parameters gradually recovered close to the normal level from 6 h after mTBI (Figures 5B,C).

### Decreased Diameter and RBC Velocity and Formation of Microthrombi of Capillaries After mTBI

**FIGURE 6:**
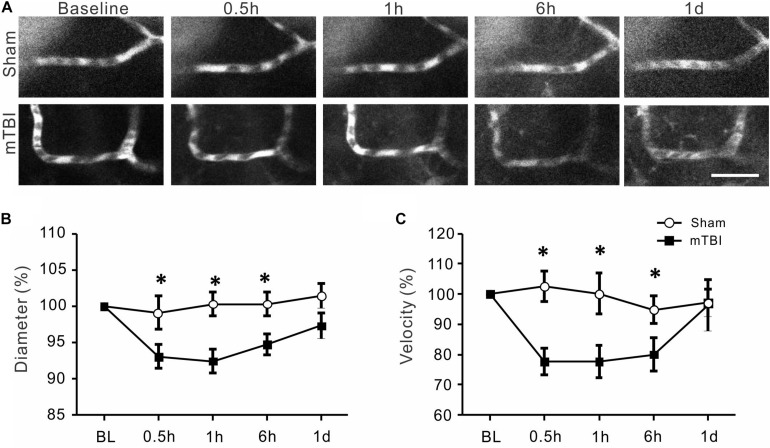
Reduced capillary diameter and RBC velocity after mTBI. **(A)** Representative images of capillary segments at different time points after mTBI of the sham and injury groups. Individual RBCs are discernable as single dark spots inside the capillaries. Scale bar: 25 μm. **(B)** Capillary diameters became smaller between 0.5 and 6 h post-injury (*p* < 0.05 at all time points), and recovered at 1 d post-mTBI (*p* > 0.05). **(C)** Changes in capillary RBC velocity were similar to that of capillary diameter: it decreased during 0.5–6 h post-mTBI and recovered at 1 d post-mTBI. *p < 0.05.

The movement of individual RBCs in capillaries were quite slow (Figure 1H, ∼0.05–0.93 mm/s) and clearly discernible under two-photon imaging (Figure 6A). While the capillaries of the sham group maintained relatively stable diameter and RBC velocity, there was a small yet significant decrease in diameters of capillaries at 0.5, 1, and 6 h after mTBI (Figure 6B. 94% ± 2, 92% ± 2, and 94% ± 1, respectively, all *p* < 0.05 when compared with the sham group at the corresponding time points), which partially recovered to baseline level at 1 day after mTBI. There were more dramatic decreases in RBC velocity of capillaries at 0.5, 1, and 6 h after mTBI (Figure 6C. 78% ± 4, 78% ± 5, 80% ± 5, respectively, all *p* < 0.05 when compared the sham group at the corresponding time points), which also increased to baseline level at 1 day after mTBI.

**FIGURE 7:**
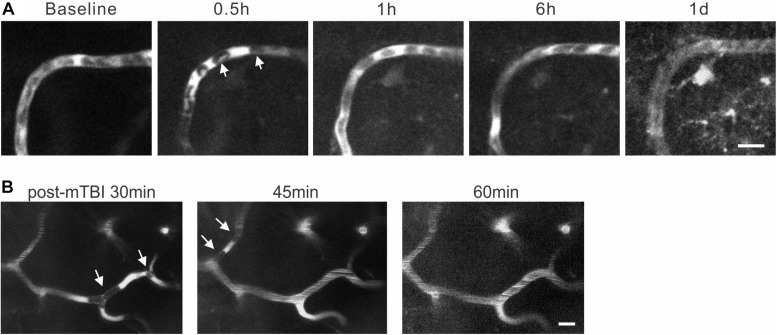
Formation and clearance of transient microthrombi following mTBI. **(A)**. At 0.5 h post-mTBI, RBCs in a segment of capillary (arrows) stopped moving and the fluorescent dye accumulated locally. This type of RBC accumulation was removed at 1 h post-mTBI and smooth blood flow recovered. **(B)** Images of cerebral capillaries between 30 and 60 min after mTBI. At 30 min, formation of microthrombi (white arrows in the left image) completely stopped blood flow in a segment of capillary. The original microthrombi disappeared at 45 min, but new ones formed (white arrows in the middle image) in different locations, which were cleared at 60 min post-mTBI (right image). Scale bars: 10 μm.

We also found that mTBI induced transient formation of microthrombi or microthrombus-like structures inside capillaries. These microthrombi consisted of a few RBCs in capillaries and partially or totally blocked blood circulation in the particular segments of capillaries. They appeared as early as 30 min after injury and sustained for a short time period (∼10–30 min) before clearance and re-perfusion of the capillaries (Figures 7A,B, *n* = 2 mice). Formation and clearance of the microthrombi occurred simultaneously at multiple locations: while a microthrombus was being cleared in one location (Figure 7B, left and middle images), a new microthrombus formed in a nearby capillary, which again disappeared in ∼15 min (Figure 7B, the middle and right images). Accompanying with microthrombosis was a reversal of blood flow direction in some capillaries, which changed back once a microthrombus was cleared.

## Discussion

In this study, we used *in vivo* two-photon microscopy in a closed-head mTBI model to study longitudinal changes in diameters and blood flow of cerebral arteries, veins, and capillaries within one day after injury. Repeated measurements of vessel diameter and centerline RBC velocity were made before and at different time points after closed-head mTBI. To our best knowledge, this is the first attempt to examine real-time longitudinal dynamics of cerebral vasculature and microcirculation at a vessel-by-vessel level in a closed-head concussion model. The technique convincingly demonstrated that cerebral arteries and capillaries had significant decreases in vessel diameter and RBC velocity from 30 min to 6 h post-mTBI and partially recovered to baseline values at one day post-mTBI. The diameter and RBC velocity of veins underwent reduction in a short time window 0.5–1 h after mTBI. We also observed formation of transient microthrombi and their subsequent clearance. These results suggest that mTBI causes acute spasm of cerebral blood vessels, reduction of blood flow, and impairment of capillary microcirculation.

We used an established closed-head mTBI model that mimics human concussion, the most common type of TBI. Consistent with published results (Schaffer et al., 2006; Drew et al., 2010b), the model induced transient loss of consciousness and impairment of motor behavior in the Rotarod test. Although the escape latency in the water maze test of the mTBI group was not significantly longer than that of the sham group, a trend of longer escape latency existed, particularly in the first 3 d after mTBI (Figure 2C), which is consistent with a previous observation of impaired spatial acquisition in 1–3 days but no change in 4–6 days after mTBI (Schaffer et al., 2006).

This model of mTBI not only mimics key features of human concussion such as closed-head injury, subtle yet diffuse structural damages, and behavioral deficits, but also is suitable for *in vivo* two-photon imaging of brain structural damage at the cellular level, including dynamics of cerebral vasculature and microcirculation. A major concern about using *in vivo* two-photon imaging for longitudinal study of mTBI is that the physical impact generated from an impact device will damage the cranial window and make it impossible to follow dynamic changes in structures of neurons, glial cells, or vasculature. By targeting the impactor tip to an area next to a small thinned skull window, we found it feasible to create an mTBI model without damaging the imaging window. We only experienced an occasional crack of window glass or leak of air into the windows. Although the physical impact from an impact device is focal, its effect on the brain is diffuse, including more distant brain regions, as was validated from our morphological data and behavioral testing results. Thus, the thinned-skull window preparation can sustain the impact of a CCI device and allow longitudinal imaging studies on dynamic changes in neuronal structures as well as vascular structures and blood circulation. This approach may also be used for testing longitudinal effects of therapeutic and rehabilitative interventions on cellular structures and activities after mTBI.

Moderate or severe TBI is known to induce a significant decrease in the cerebral blood flow (CBF) in patients during the acute stage (less than 3 h after head injury) and chronic stage (at least 3 months after TBI), as measured by transcranial Doppler (TCD) ultrasound or arterial spin labeling perfusion fMRI techniques (McQuire et al., 1998; Shi et al., 2010). Cerebral hypoperfusion also appears in rat TBI models with severe damage in the early stage (4 h after cortical contusion) and late stage (9 months after lateral fluid-percussion injury) using arterial spin labeling (ASL) MRI technique or Laser Doppler Flowmetry (LDF) measurement (Thomale et al., 2002; Hayward et al., 2010, 2011). Besides lower CBF, reduction in carotid artery blood flow was also observed after a rapid head rotational injury (Clevenger et al., 2015).

Reduced CBF after mTBI is observed in both animal and human studies. In acute sports-related concussion patients, ASL MRI techniques showed decreased CBF in several brain regions in 24–48 h after injury (Wang et al., 2018). Furthermore, the decrease in CBF in 8 d after injury was more severe and diffuse than at 24 h after injury (Wang et al., 2016). In a rat model of blast-induced mild TBI, a reduction in cerebral perfusion was found with LDF within 2 h after injury (Kawoos et al., 2016). In a mouse closed-head mTBI model, a single concussion caused CBF reduction by 35 ± 4% at 4 h, which returned to preinjury levels by 24 h (Buckley et al., 2015). These results are generally consistent with our observation that RBC velocity was decreased between 30 min to 6 h after mTBI, before recovering to about baseline level at 24 h. In contrast, an investigation on 14 patients with mTBI using an ASL technique showed increases in regional CBF in the striatum and frontal and occipital cortex in 3 h to 10 days after injury, which was accompanied by high level of oxygen in veins (Doshi et al., 2015). Similarly, increases in cerebral blood flow and pial arteriolar diameter were observed in a fluid percussion model of TBI (Wei et al., 1980, 1981). This discrepancy may originate from the wide range of post-concussion time periods of the patients in that human study (Doshi et al., 2015), or from the difference in injury model and severity as well as animal species (Wei et al., 1980, 1981). Although our results showed recovery of vessel diameter and RBC velocity to baseline level at 1 day after mTBI, such recovery may not be complete and stable. Changes in chronic post-concussion period need to be further determined in future.

There is apparent discrepancy between changes in the diameters and velocities in arterials and venous (Figures 4, 5). Because many other factors also contribute to the blood flow volume, including numbers and lengths of arteries and veins, velocity changes, and extracellular space, it would be difficult to estimate the volume of blood flow by imaging diameter and velocity of a small numbers of vessels of the brain. On the other hand, an assumption that total inflow of blood should equal to outflow may not be true, particularly under pathological conditions such as TBI. Disturbed cerebral microcirculation participated in the pathological process of neurological disease; changes in diameter and velocity of arteries and veins can be quite different in various pathological conditions. For example, in 60 min after subarachnoid hemorrhage, arterioles become dilated but venules do not change (Ishikawa et al., 2016). In diabetic mice, there was no significant change in the blood velocity of arterioles while there was a significant decline in those of venules (Huang et al., 2014). Middle cerebral artery occlusion in diabetic mice can cause the blood velocities of both arterioles and venules to decrease (Huang et al., 2014). Changes of velocities and diameters of arterioles and venules also showed potential discrepancy in recovery one day after mTBI induction in the present study. Gattinoni et al. have proposed that venous and arterial base excess difference is closely related to physiological reality (Lang and Zander, 2002; Gattinoni and Busana, 2019).

The present study showed that the mean RBC velocity in all animals at baseline was 0.25 mm/s (0.05–0.93 mm/s) and the mean value from each animal varied from 0.10 to 0.44 mm/s. In contrast, the mean capillary RBC velocities were within the scopes of 0.1∼1.6 mm/s and 0.05∼0.9 mm/s as reported by Emmanuelle (Chaigneau et al., 2003) and Stefanovic et al. (2008). In some other studies, the velocities in both anesthetized and awake mice were calculated to be approximately 0.7 or 1.3 mm/s (Huang et al., 2014; Wei et al., 2016; Li et al., 2019; Lu et al., 2019). Comparing to these values, our data seemed to be lower. One possible reason may be due to the relatively smaller capillaries (with a mean diameter of 3.96 μm) we imaged than those imaged in prior studies (with mean diameters of 4.7 to 6 μm) (Li et al., 2019; Lu et al., 2019). Small vessels usually have lower RBC velocity. Another possible reason may be due to different effects of anesthetic agents on blood vessel diameters (Slupe and Kirsch, 2018). For example, ketamine has been shown to cause constriction of microvessels (Small et al., 2014).

The mechanism of mTBI-induced decrease in cerebral blood flow may involve vasospasm, edema, and neuroinflammation. A body of studies have reported post-TBI vasospasm (Kramer et al., 2013; Fehnel et al., 2014; Perrein et al., 2015; Ogami et al., 2017). In severe TBI, post- traumatic vasospasm is detected in 30–40% of patients (Perrein et al., 2015). Vasospasm appears in the early stage after TBI and is considered related to delayed ischemic neurological deficit, which contributes to the formation of subarachnoid hemorrhage (DeWitt and Prough, 2003). In the present study, direct measurement of decreased arteriole diameter between 0.5 and 6 h post-mTBI provides strong evidence of vasospasm during the early time period after the injury. A decrease in vein diameter after mTBI was also observed. Since the diameters of cerebral arteries and veins are positively correlated to their RBC velocity (Figures 1F–H), the decreased vessel diameters after mTBI likely contribute to the reduced CBF. Mechanistically, calponin phosphorylation and carbon dioxide (Pa CO_2_) may be factors involved in regulation of cerebrovascular reactivity to TBI (Shen et al., 2007; Len et al., 2011). Increases in the level of NO and Ca^++^ in endothelium after TBI are also considered major factors in stimulating vasospasm of cerebral arteries (Villalba et al., 2014). In addition, we also observed early reductions of diameters and RBC velocity of capillaries in 0.5–6 h after mild TBI in our study. Because cerebral blood flows in the capillary and arteriole are regulated through different signaling cascades (Biesecker et al., 2016; Mishra et al., 2016), it would be interesting to investigate whether different molecular mechanisms are involved in the regulation of blood flow in arteries and capillaries after mTBI.

Edema is known to develop after moderate to severe TBI and contribute to increased intracranial pressure (ICP) (Bolouri et al., 2012). An increase of ICP from tissue edema is thought to be one of the major mechanisms of blood flow loss (Nortje and Menon, 2004). However, increase in ICP after TBI varies considerably in different TBI models as well as in different animal species (Reid et al., 2010). Particularly, TBI alone does not cause significant increase of ICP in either lateral fluid percussion or impact acceleration induced injury in rats (Gabrielian et al., 2011) and closed-head mTBI is shown not to cause edema in a weight drop model in mice (Garcia et al., 1994; Kane et al., 2012). Therefore, edema may not make a significant contribution to the reduced RBC velocity in our mTBI mice.

Microthrombosis resulting from abnormal platelet activation occurs in the early stage after moderate and severe TBI, which can lead to perfusion loss in the peri-contusional cortex (Dietrich et al., 1994; Maeda et al., 1997; Bramlett and Dietrich, 2004). There is a positive correlation between the number of microthrombi and the degree of neuronal necrosis (Stein et al., 2004). In the present study, the observed microthrombi in capillaries seem different from typical microthrombi that occur after more severe TBI. Among all the capillaries being imaged, we observed only three cases of microthrombus formation in the mTBI brain and none in sham brain. Given the rare and transient nature of microthrombosis in this particular model of mTBI, it was not possible to quantify them. These capillary microthrombi were transient and reversible, lasting for only minutes to tens of minutes. They may be regarded as a mild type of microthrombi. Given the transient and microscopic nature of the microthrombi, it would be difficult to capture them using conventional imaging techniques such as CT scan or even tissue histology. Their pathological significance is unclear. In controlled cortical impact and fluid percussion models of TBI, factors that may induce or facilitate microthrombosis include endothelial damage and release of procoagulant molecules such as cardiolipin (Kontos et al., 1981; Zhao et al., 2016; Hall et al., 2017), and release of platelet activating factor and platelet aggregation (Rosenblum et al., 1982; Maeda et al., 1997). While a high density of microthrombi is associate with more severe selective neuronal necrosis (Deng et al., 2005), the transient microthrombi we observed after mTBI may not be severe enough to cause neuronal death. However, their interference with capillary microcirculation likely contributes to exacerbating brain ischemia and acute functional deficits.

In summary, we used an *in vivo* two-photon imaging technique to study acute changes in cerebral vasculature and blood circulation in a model of closed-head mTBI. Our results provide direct evidence that mTBI caused acute decreases in the diameter and blood flow of cerebral arterioles, veins, and capillaries to different degrees within 0.5 to 6 h after injury. These decreases mostly recovered to baseline levels at 24 h after injury. The mTBI also caused microthrombosis in capillaries, which were transient and cleared in a short time period. These vascular changes may contribute to acute brain ischemia and functional impairments and may guide therapeutic development for mTBI.

## Data Availability Statement

All datasets generated for this study are included in the article/supplementary material.

## Ethics Statement

The animal study was reviewed and approved by the Institutional Animal Care and Use Committee (IACUC) of the Indiana University School of Medicine.

## Author Contributions

XJ and YR conceived and designed the experiments. XH and XP performed the experiments:. XH, XP, ZC, L-JS, and CM analyzed the data. XH, ZC, L-JS, CM, XJ, and YR wrote, revised, and commented on the manuscript.

## Conflict of Interest

The authors declare that the research was conducted in the absence of any commercial or financial relationships that could be construed as a potential conflict of interest.
